# Diethyl 4-(4-acetamido­phenyl)-2,6-dimethyl-1,4-dihydro­pyridine-3,5-dicarboxyl­ate

**DOI:** 10.1107/S160053681202750X

**Published:** 2012-07-25

**Authors:** Yiliang Zhao, David E. Hibbs, Paul W. Groundwater, Abram Wassef

**Affiliations:** a Faculty of Pharmacy, The University of Sydney, NSW 2006, Australia

## Abstract

The title compound, C_21_H_26_N_2_O_5_, was unexpectedly obtained as a by-product in the reaction of ethyl acetoacetate, 4-acetamido­benzaldehyde and urea under microwave irradiation. The dihydro­pyridine ring assumes a flattened boat conformation. Inter­molecular N—H⋯O and weak C—H⋯O hydrogen bonding occurs in the crystal.

## Related literature

For the Biginelli dihydro­pyrimidone and Hantzsch dihydro­pyridine syntheses, see: Kappe & Stadler (2004 ▶); Kumar & Maurya (2008 ▶). For the microwave synthesis and melting point of 4-(3-acetamido­phen­yl)-6-methyl-2-oxo-1,2,3,4-tetra­hydro­pyrimidine-5-carboxyl­ate, see: Mobinikhaledi & Foroughifar (2006 ▶).
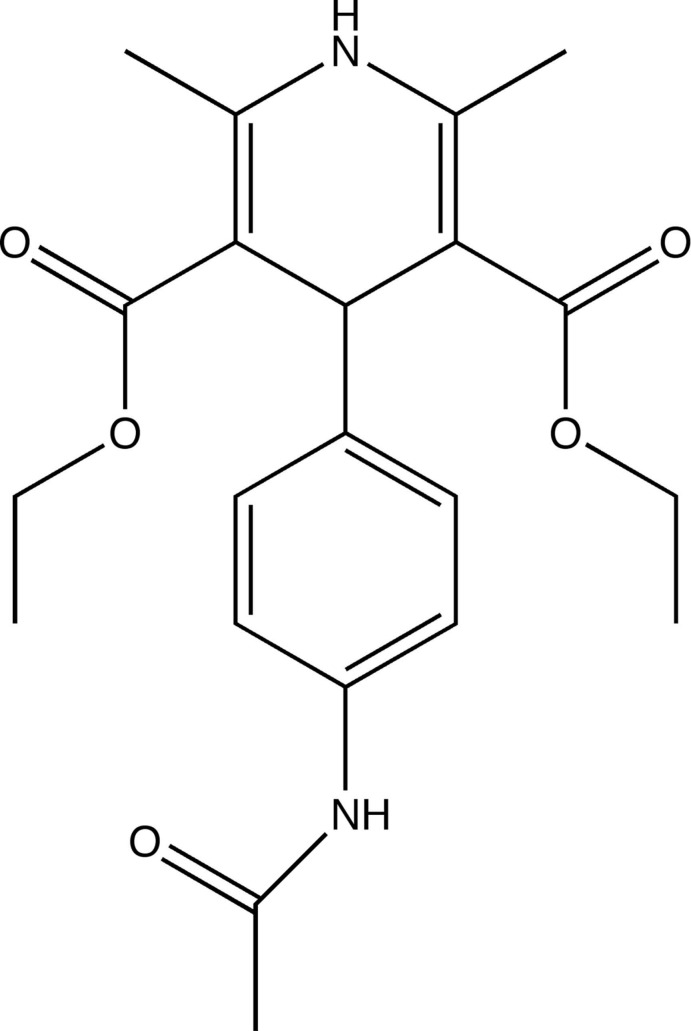


## Experimental

### 

#### Crystal data

C_21_H_26_N_2_O_5_
*M* *_r_* = 386.44
Monoclinic, 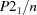

*a* = 11.3359 (7) Å
*b* = 12.1934 (7) Å
*c* = 15.3262 (9) Å
β = 109.745 (1)°
*V* = 1993.9 (2) Å^3^
*Z* = 4
Mo *K*α radiation
μ = 0.09 mm^−1^
*T* = 150 K
0.2 × 0.2 × 0.15 mm

#### Data collection

Bruker APEXII CCD area-detector diffractometer
Absorption correction: multi-scan (*SADABS*; Bruker, 2001 ▶) *T*_min_ = 0.976, *T*_max_ = 0.988
15313 measured reflections
4593 independent reflections
3602 reflections with *I* > 2σ(*I*)
*R*_int_ = 0.027

#### Refinement

*R*[*F* ^2^ > 2σ(*F* ^2^)] = 0.043
*wR*(*F* ^2^) = 0.120
*S* = 1.05
4593 reflections
266 parameters
H atoms treated by a mixture of independent and constrained refinement
Δρ_max_ = 0.28 e Å^−3^
Δρ_min_ = −0.20 e Å^−3^

### 

Data collection: *APEX2* (Bruker, 2007 ▶); cell refinement: *SAINT* (Bruker, 2007 ▶); data reduction: *SAINT*; program(s) used to solve structure: *SHELXS97* (Sheldrick, 2008 ▶); program(s) used to refine structure: *SHELXL97* (Sheldrick, 2008 ▶); molecular graphics: *OLEX2* (Dolomanov *et al.*, 2009 ▶); software used to prepare material for publication: *OLEX2*.

## Supplementary Material

Crystal structure: contains datablock(s) I, global. DOI: 10.1107/S160053681202750X/xu5556sup1.cif

Structure factors: contains datablock(s) I. DOI: 10.1107/S160053681202750X/xu5556Isup2.hkl

Supplementary material file. DOI: 10.1107/S160053681202750X/xu5556Isup3.cml

Additional supplementary materials:  crystallographic information; 3D view; checkCIF report

## Figures and Tables

**Table 1:** Hydrogen-bond geometry (Å, °)

*D*—H⋯*A*	*D*—H	H⋯*A*	*D*⋯*A*	*D*—H⋯*A*
N1—H1⋯O5^i^	0.892 (19)	2.022 (19)	2.8946 (17)	165.5 (16)
N2—H2⋯O3^ii^	0.866 (19)	2.074 (19)	2.9383 (17)	175.4 (16)
C6—H6*A*⋯O1^iii^	0.98	2.52	3.370 (2)	145
C13—H13*A*⋯O1^iv^	0.98	2.38	3.337 (2)	165

Symmetry codes: (i) 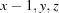; (ii) 
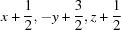
; (iii) 
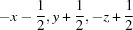
; (iv) 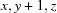.

**Fig. 1.**
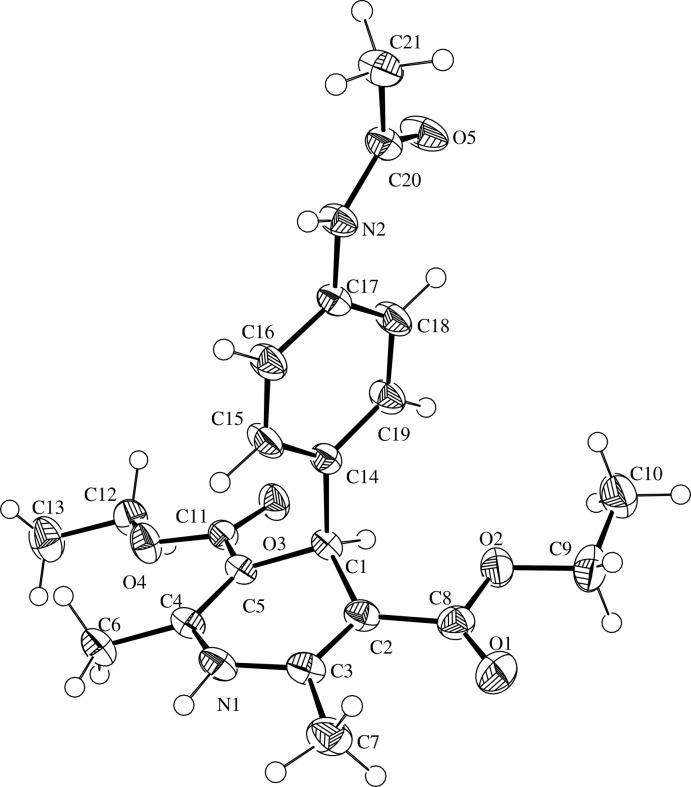
The molecular structure of the title compound with 50% probability displacement ellipsoids for non-H atoms.

## supplementary crystallographic information

### Comment

The bond lengths and angles in the title compound are as expected. The maximum deviation from the mean plane of the central ring is 0.188 (1) A for C1. The C8 and C11 carboxylate side chains are essentially planar, with a maximum deviation of 0.077 A for C10. These groups are set at 11.64 (8)° and 25.65 (4)° to the central ring plane respectively.

### Experimental

A mixture of 4-acetamidobenzaldehyde (163 mg, 1 mmol), ethyl acetoacetate (130 mg, 1 mmol) and urea (60 mg, 1 mmol) was stirred for 2 h at 403 K under microwave irradiation. After completion of the reaction, TLC showed the presence of two main products, which were separated using silica chromatography, eluting with dichloromthane/methanol to give diethyl 4-acetamidophenyl-1,4-dihydro-2,6-dimethylpyridine-3,5-dicarboxylate (120 mg, 31%, mp 254–256°C), and ethyl 4-(3-acetamidophenyl)-6-methyl-2-oxo-1,2,3,4-tetrahydropyrimidine-5-carboxylate (72 mg, 23%, mp 284–286°C).

### Refinement

Amino H atoms were located in a difference Fourier map and refined isotropically. Other H atoms were placed in calculated positions with C—H = 0.95 to 1.00 Å, and were refined in a riding mode with *U*_iso_(H) = 1.5*U*_eq_(C) for methyl H atoms and 1.2*U*_eq_(C) for the others.

### Crystal data

**Table:**

C_21_H_26_N_2_O_5_	*D*_x_ = 1.287 Mg m^−^^3^
*M**_r_* = 386.44	Melting point: 256 K
Monoclinic, *P*2_1_/*n*	Mo *K*α radiation, λ = 0.71073 Å
*a* = 11.3359 (7) Å	Cell parameters from 15313 reflections
*b* = 12.1934 (7) Å	θ = 2.0–28.3°
*c* = 15.3262 (9) Å	µ = 0.09 mm^−^^1^
β = 109.745 (1)°	*T* = 150 K
*V* = 1993.9 (2) Å^3^	Block, clear colourless
*Z* = 4	0.2 × 0.2 × 0.15 mm
*F*(000) = 824	

### Data collection

**Table:**

Bruker APEXII CCD area-detector diffractometer	4593 independent reflections
Radiation source: sealed tube	3602 reflections with *I* > 2σ(*I*)
Graphite monochromator	*R*_int_ = 0.027
Detector resolution: 8 pixels mm^-1^	θ_max_ = 28.3°, θ_min_ = 2.0°
ω and φ scans	*h* = −15→14
Absorption correction: multi-scan (*SADABS*; Bruker, 2001)	*k* = −15→14
*T*_min_ = 0.976, *T*_max_ = 0.988	*l* = −18→19
15313 measured reflections	

### Refinement

**Table:**

Refinement on *F*^2^	Primary atom site location: structure-invariant direct methods
Least-squares matrix: full	Secondary atom site location: difference Fourier map
*R*[*F*^2^ > 2σ(*F*^2^)] = 0.043	Hydrogen site location: inferred from neighbouring sites
*wR*(*F*^2^) = 0.120	H atoms treated by a mixture of independent and constrained refinement
*S* = 1.05	*w* = 1/[σ^2^(*F*_o_^2^) + (0.0575*P*)^2^ + 0.5603*P*] where *P* = (*F*_o_^2^ + 2*F*_c_^2^)/3
4593 reflections	(Δ/σ)_max_ < 0.001
266 parameters	Δρ_max_ = 0.28 e Å^−^^3^
0 restraints	Δρ_min_ = −0.20 e Å^−^^3^

### Special details

**Table:**

Geometry. All e.s.d.'s (except the e.s.d. in the dihedral angle between two l.s. planes) are estimated using the full covariance matrix. The cell e.s.d.'s are taken into account individually in the estimation of e.s.d.'s in distances, angles and torsion angles; correlations between e.s.d.'s in cell parameters are only used when they are defined by crystal symmetry. An approximate (isotropic) treatment of cell e.s.d.'s is used for estimating e.s.d.'s involving l.s. planes.
Refinement. Refinement of *F*^2^ against ALL reflections. The weighted *R*-factor *wR* and goodness of fit *S* are based on *F*^2^, conventional *R*-factors *R* are based on *F*, with *F* set to zero for negative *F*^2^. The threshold expression of *F*^2^ > σ(*F*^2^) is used only for calculating *R*-factors(gt) *etc*. and is not relevant to the choice of reflections for refinement. *R*-factors based on *F*^2^ are statistically about twice as large as those based on *F*, and *R*- factors based on ALL data will be even larger.

### Fractional atomic coordinates and isotropic or equivalent isotropic displacement parameters (Å^2^)

**Table:**

	*x*	*y*	*z*	*U*_iso_*/*U*_eq_	
O5	0.66512 (9)	0.67514 (10)	0.46869 (8)	0.0399 (3)	
O2	0.05682 (10)	0.56346 (9)	0.19250 (8)	0.0364 (3)	
N1	−0.12494 (11)	0.70015 (10)	0.40319 (9)	0.0314 (3)	
H1	−0.1795 (16)	0.6891 (14)	0.4328 (12)	0.037 (5)*	
O3	0.03829 (10)	0.94192 (9)	0.23298 (7)	0.0334 (3)	
O4	−0.04968 (10)	1.01757 (8)	0.32887 (7)	0.0345 (3)	
C13	−0.10344 (19)	1.20435 (14)	0.33222 (14)	0.0474 (4)	
H13A	−0.1056	1.2765	0.3036	0.071*	
H13B	−0.0550	1.2090	0.3985	0.071*	
H13C	−0.1891	1.1807	0.3241	0.071*	
N2	0.53871 (11)	0.64292 (10)	0.55364 (9)	0.0285 (3)	
H2	0.5381 (15)	0.6217 (14)	0.6074 (13)	0.037 (5)*	
C1	0.03998 (12)	0.72957 (11)	0.30406 (10)	0.0249 (3)	
H1A	0.0423	0.7441	0.2404	0.030*	
C2	−0.03113 (12)	0.62288 (12)	0.30116 (10)	0.0281 (3)	
O1	−0.05548 (11)	0.44057 (10)	0.23947 (9)	0.0455 (3)	
C3	−0.10251 (13)	0.61059 (12)	0.35593 (11)	0.0304 (3)	
C4	−0.10146 (12)	0.80695 (12)	0.38358 (10)	0.0274 (3)	
C20	0.65040 (13)	0.64295 (12)	0.54023 (10)	0.0291 (3)	
C17	0.41948 (12)	0.67243 (11)	0.48949 (10)	0.0259 (3)	
C18	0.39430 (13)	0.67984 (14)	0.39484 (11)	0.0334 (3)	
H18	0.4597	0.6700	0.3699	0.040*	
C19	0.27293 (13)	0.70175 (13)	0.33631 (10)	0.0312 (3)	
H19	0.2568	0.7066	0.2714	0.037*	
C14	0.17503 (12)	0.71665 (11)	0.36981 (10)	0.0248 (3)	
C8	−0.01382 (13)	0.53303 (13)	0.24354 (10)	0.0318 (3)	
C9	0.08124 (17)	0.47931 (15)	0.13380 (12)	0.0435 (4)	
H9A	0.0033	0.4608	0.0825	0.052*	
H9B	0.1134	0.4120	0.1702	0.052*	
C10	0.17680 (18)	0.52463 (18)	0.09623 (14)	0.0540 (5)	
H10A	0.1950	0.4704	0.0553	0.081*	
H10B	0.2538	0.5415	0.1476	0.081*	
H10C	0.1443	0.5917	0.0611	0.081*	
C6	−0.16499 (14)	0.89009 (13)	0.42459 (11)	0.0348 (3)	
H6A	−0.2245	0.9327	0.3747	0.052*	
H6B	−0.1021	0.9394	0.4654	0.052*	
H6C	−0.2099	0.8525	0.4604	0.052*	
C5	−0.02787 (12)	0.82421 (11)	0.33070 (9)	0.0251 (3)	
C16	0.32309 (13)	0.69036 (14)	0.52466 (10)	0.0336 (3)	
H16	0.3397	0.6877	0.5897	0.040*	
C15	0.20282 (13)	0.71204 (14)	0.46516 (10)	0.0339 (3)	
H15	0.1378	0.7240	0.4902	0.041*	
C21	0.75834 (14)	0.59981 (14)	0.61983 (11)	0.0377 (4)	
H21A	0.7863	0.5297	0.6023	0.057*	
H21B	0.7316	0.5889	0.6737	0.057*	
H21C	0.8275	0.6526	0.6354	0.057*	
C11	−0.00926 (12)	0.93072 (12)	0.29325 (10)	0.0263 (3)	
C12	−0.04350 (15)	1.12340 (12)	0.28729 (12)	0.0353 (3)	
H12A	−0.0888	1.1209	0.2196	0.042*	
H12B	0.0448	1.1440	0.2980	0.042*	
C7	−0.16252 (16)	0.50641 (13)	0.37219 (13)	0.0410 (4)	
H7A	−0.0973	0.4542	0.4058	0.062*	
H7B	−0.2130	0.4747	0.3125	0.062*	
H7C	−0.2165	0.5223	0.4089	0.062*	

### Atomic displacement parameters (Å^2^)

**Table:**

	*U*^11^	*U*^22^	*U*^33^	*U*^12^	*U*^13^	*U*^23^
O5	0.0260 (5)	0.0586 (8)	0.0404 (6)	0.0018 (5)	0.0182 (5)	0.0136 (5)
O2	0.0400 (6)	0.0352 (6)	0.0377 (6)	0.0008 (5)	0.0181 (5)	−0.0058 (5)
N1	0.0273 (6)	0.0348 (7)	0.0380 (7)	0.0011 (5)	0.0188 (5)	0.0062 (5)
O3	0.0403 (6)	0.0334 (6)	0.0346 (6)	−0.0017 (4)	0.0234 (5)	−0.0011 (4)
O4	0.0455 (6)	0.0290 (6)	0.0376 (6)	0.0042 (4)	0.0255 (5)	0.0030 (4)
C13	0.0610 (11)	0.0339 (9)	0.0562 (11)	0.0067 (8)	0.0315 (9)	0.0010 (8)
N2	0.0233 (6)	0.0373 (7)	0.0277 (6)	0.0016 (5)	0.0125 (5)	0.0060 (5)
C1	0.0226 (6)	0.0274 (7)	0.0273 (7)	0.0024 (5)	0.0117 (5)	0.0016 (5)
C2	0.0241 (6)	0.0275 (7)	0.0326 (8)	0.0015 (5)	0.0096 (6)	0.0035 (6)
O1	0.0494 (7)	0.0350 (7)	0.0528 (8)	−0.0080 (5)	0.0181 (6)	−0.0071 (5)
C3	0.0246 (7)	0.0304 (8)	0.0364 (8)	0.0011 (6)	0.0106 (6)	0.0051 (6)
C4	0.0222 (6)	0.0315 (8)	0.0299 (7)	0.0035 (5)	0.0109 (5)	0.0038 (6)
C20	0.0247 (7)	0.0307 (7)	0.0343 (8)	−0.0003 (5)	0.0132 (6)	0.0021 (6)
C17	0.0226 (6)	0.0274 (7)	0.0301 (7)	0.0009 (5)	0.0120 (5)	0.0031 (6)
C18	0.0255 (7)	0.0487 (9)	0.0314 (8)	0.0036 (6)	0.0168 (6)	0.0028 (7)
C19	0.0279 (7)	0.0427 (9)	0.0265 (7)	0.0022 (6)	0.0138 (6)	0.0036 (6)
C14	0.0236 (6)	0.0238 (7)	0.0297 (7)	0.0005 (5)	0.0125 (5)	0.0014 (5)
C8	0.0275 (7)	0.0323 (8)	0.0325 (8)	0.0005 (6)	0.0059 (6)	0.0007 (6)
C9	0.0536 (10)	0.0402 (9)	0.0345 (9)	0.0071 (8)	0.0122 (8)	−0.0084 (7)
C10	0.0478 (10)	0.0692 (13)	0.0484 (11)	0.0072 (9)	0.0207 (9)	−0.0147 (10)
C6	0.0343 (8)	0.0382 (9)	0.0394 (9)	0.0051 (6)	0.0224 (7)	0.0031 (7)
C5	0.0211 (6)	0.0281 (7)	0.0276 (7)	0.0022 (5)	0.0102 (5)	0.0016 (5)
C16	0.0284 (7)	0.0504 (10)	0.0258 (7)	0.0036 (6)	0.0145 (6)	0.0031 (6)
C15	0.0250 (7)	0.0510 (10)	0.0316 (8)	0.0053 (6)	0.0172 (6)	0.0013 (7)
C21	0.0269 (7)	0.0478 (10)	0.0389 (9)	0.0038 (6)	0.0117 (6)	0.0054 (7)
C11	0.0231 (6)	0.0310 (7)	0.0256 (7)	0.0009 (5)	0.0095 (5)	−0.0014 (6)
C12	0.0414 (8)	0.0292 (8)	0.0405 (9)	0.0000 (6)	0.0207 (7)	0.0028 (7)
C7	0.0382 (8)	0.0350 (9)	0.0555 (10)	−0.0041 (7)	0.0233 (8)	0.0058 (8)

### Geometric parameters (Å, º)

**Table:**

O5—C20	1.2278 (18)	C17—C16	1.3894 (19)
O2—C8	1.3469 (19)	C18—H18	0.9500
O2—C9	1.4515 (19)	C18—C19	1.391 (2)
N1—H1	0.893 (18)	C19—H19	0.9500
N1—C3	1.381 (2)	C19—C14	1.3833 (19)
N1—C4	1.3824 (19)	C14—C15	1.388 (2)
O3—C11	1.2244 (17)	C9—H9A	0.9900
O4—C11	1.3410 (17)	C9—H9B	0.9900
O4—C12	1.4513 (18)	C9—C10	1.494 (3)
C13—H13A	0.9800	C10—H10A	0.9800
C13—H13B	0.9800	C10—H10B	0.9800
C13—H13C	0.9800	C10—H10C	0.9800
C13—C12	1.492 (2)	C6—H6A	0.9800
N2—H2	0.867 (18)	C6—H6B	0.9800
N2—C20	1.3495 (18)	C6—H6C	0.9800
N2—C17	1.4230 (18)	C5—C11	1.464 (2)
C1—H1A	1.0000	C16—H16	0.9500
C1—C2	1.5231 (19)	C16—C15	1.386 (2)
C1—C14	1.5303 (18)	C15—H15	0.9500
C1—C5	1.5173 (18)	C21—H21A	0.9800
C2—C3	1.357 (2)	C21—H21B	0.9800
C2—C8	1.461 (2)	C21—H21C	0.9800
O1—C8	1.2157 (19)	C12—H12A	0.9900
C3—C7	1.501 (2)	C12—H12B	0.9900
C4—C6	1.499 (2)	C7—H7A	0.9800
C4—C5	1.3615 (19)	C7—H7B	0.9800
C20—C21	1.501 (2)	C7—H7C	0.9800
C17—C18	1.384 (2)		
			
C8—O2—C9	116.13 (13)	O2—C9—H9B	110.3
C3—N1—H1	115.4 (11)	O2—C9—C10	107.00 (15)
C3—N1—C4	123.22 (12)	H9A—C9—H9B	108.6
C4—N1—H1	118.1 (11)	C10—C9—H9A	110.3
C11—O4—C12	116.72 (11)	C10—C9—H9B	110.3
H13A—C13—H13B	109.5	C9—C10—H10A	109.5
H13A—C13—H13C	109.5	C9—C10—H10B	109.5
H13B—C13—H13C	109.5	C9—C10—H10C	109.5
C12—C13—H13A	109.5	H10A—C10—H10B	109.5
C12—C13—H13B	109.5	H10A—C10—H10C	109.5
C12—C13—H13C	109.5	H10B—C10—H10C	109.5
C20—N2—H2	117.1 (11)	C4—C6—H6A	109.5
C20—N2—C17	128.01 (13)	C4—C6—H6B	109.5
C17—N2—H2	114.9 (11)	C4—C6—H6C	109.5
C2—C1—H1A	108.1	H6A—C6—H6B	109.5
C2—C1—C14	109.46 (11)	H6A—C6—H6C	109.5
C14—C1—H1A	108.1	H6B—C6—H6C	109.5
C5—C1—H1A	108.1	C4—C5—C1	120.71 (12)
C5—C1—C2	110.25 (11)	C4—C5—C11	124.68 (13)
C5—C1—C14	112.73 (11)	C11—C5—C1	114.59 (11)
C3—C2—C1	119.78 (13)	C17—C16—H16	119.9
C3—C2—C8	121.40 (13)	C15—C16—C17	120.17 (13)
C8—C2—C1	118.69 (12)	C15—C16—H16	119.9
N1—C3—C7	114.09 (13)	C14—C15—H15	119.2
C2—C3—N1	119.42 (13)	C16—C15—C14	121.56 (13)
C2—C3—C7	126.49 (14)	C16—C15—H15	119.2
N1—C4—C6	113.00 (12)	C20—C21—H21A	109.5
C5—C4—N1	118.48 (13)	C20—C21—H21B	109.5
C5—C4—C6	128.51 (13)	C20—C21—H21C	109.5
O5—C20—N2	123.61 (13)	H21A—C21—H21B	109.5
O5—C20—C21	121.22 (13)	H21A—C21—H21C	109.5
N2—C20—C21	115.17 (13)	H21B—C21—H21C	109.5
C18—C17—N2	123.52 (12)	O3—C11—O4	121.14 (13)
C18—C17—C16	119.06 (13)	O3—C11—C5	123.59 (13)
C16—C17—N2	117.37 (13)	O4—C11—C5	115.26 (12)
C17—C18—H18	120.1	O4—C12—C13	106.77 (13)
C17—C18—C19	119.83 (13)	O4—C12—H12A	110.4
C19—C18—H18	120.1	O4—C12—H12B	110.4
C18—C19—H19	119.0	C13—C12—H12A	110.4
C14—C19—C18	121.91 (13)	C13—C12—H12B	110.4
C14—C19—H19	119.0	H12A—C12—H12B	108.6
C19—C14—C1	121.24 (12)	C3—C7—H7A	109.5
C19—C14—C15	117.40 (13)	C3—C7—H7B	109.5
C15—C14—C1	121.21 (12)	C3—C7—H7C	109.5
O2—C8—C2	111.84 (13)	H7A—C7—H7B	109.5
O1—C8—O2	121.51 (14)	H7A—C7—H7C	109.5
O1—C8—C2	126.65 (15)	H7B—C7—H7C	109.5
O2—C9—H9A	110.3		
			
C5—C1—C2—C3	−28.32 (18)	C9—O2—C8—C2	179.07 (12)
C14—C1—C2—C3	96.23 (15)	C3—C2—C8—O1	−2.2 (2)
C5—C1—C2—C8	155.75 (12)	C1—C2—C8—O1	173.71 (14)
C14—C1—C2—C8	−79.70 (15)	C3—C2—C8—O2	178.01 (13)
C8—C2—C3—N1	−174.57 (13)	C1—C2—C8—O2	−6.13 (18)
C1—C2—C3—N1	9.6 (2)	C8—O2—C9—C10	−170.92 (13)
C8—C2—C3—C7	5.6 (2)	N1—C4—C5—C11	170.85 (13)
C1—C2—C3—C7	−170.23 (14)	C6—C4—C5—C11	−8.3 (2)
C4—N1—C3—C2	14.1 (2)	N1—C4—C5—C1	−7.6 (2)
C4—N1—C3—C7	−166.00 (13)	C6—C4—C5—C1	173.31 (14)
C3—N1—C4—C5	−15.2 (2)	C2—C1—C5—C4	27.40 (18)
C3—N1—C4—C6	164.06 (13)	C14—C1—C5—C4	−95.25 (15)
C17—N2—C20—O5	3.8 (3)	C2—C1—C5—C11	−151.16 (12)
C17—N2—C20—C21	−175.95 (14)	C14—C1—C5—C11	86.19 (14)
C20—N2—C17—C18	17.1 (2)	C18—C17—C16—C15	1.9 (2)
C20—N2—C17—C16	−165.46 (15)	N2—C17—C16—C15	−175.64 (15)
C16—C17—C18—C19	−1.9 (2)	C17—C16—C15—C14	−0.1 (3)
N2—C17—C18—C19	175.51 (14)	C19—C14—C15—C16	−1.8 (2)
C17—C18—C19—C14	0.0 (2)	C1—C14—C15—C16	174.01 (14)
C18—C19—C14—C15	1.8 (2)	C12—O4—C11—O3	4.8 (2)
C18—C19—C14—C1	−173.97 (14)	C12—O4—C11—C5	−174.40 (12)
C5—C1—C14—C19	−133.47 (14)	C4—C5—C11—O3	−166.46 (14)
C2—C1—C14—C19	103.43 (15)	C1—C5—C11—O3	12.0 (2)
C5—C1—C14—C15	50.92 (18)	C4—C5—C11—O4	12.8 (2)
C2—C1—C14—C15	−72.17 (17)	C1—C5—C11—O4	−168.74 (11)
C9—O2—C8—O1	−0.8 (2)	C11—O4—C12—C13	175.03 (14)

### Hydrogen-bond geometry (Å, º)

**Table:**

*D*—H···*A*	*D*—H	H···*A*	*D*···*A*	*D*—H···*A*
N1—H1···O5^i^	0.892 (19)	2.022 (19)	2.8946 (17)	165.5 (16)
N2—H2···O3^ii^	0.866 (19)	2.074 (19)	2.9383 (17)	175.4 (16)
C6—H6*A*···O1^iii^	0.98	2.52	3.370 (2)	145
C13—H13*A*···O1^iv^	0.98	2.38	3.337 (2)	165

Symmetry codes: (i) *x*−1, *y*, *z*; (ii) *x*+1/2, −*y*+3/2, *z*+1/2; (iii) −*x*−1/2, *y*+1/2, −*z*+1/2; (iv) *x*, *y*+1, *z*.
